# Menouria due to congenital vesicovaginal fistula associated with complex genitourinary malformation

**DOI:** 10.4103/0970-1591.57924

**Published:** 2009

**Authors:** N. Rajamaheshwari, K. Seethalakshmi, Lily Varghese

**Affiliations:** Department of Urogynecology, Government Kasturba Gandhi, Hospital, Madras Medical College, Triplicane, Chennai - 600 005, Tamil Nadu, India; 1 Institute of Social Obstetrics, Government Kasturba Gandhi, Hospital, Madras Medical College, Triplicane, Chennai - 600 005, Tamil Nadu, India

**Keywords:** Congenital vesicovaginal fistula, menouria, transverse vaginal septum, unilateral renal agenesis

## Abstract

**Background:**

Congenital vesicovaginal fistula (VVF) is a very uncommon condition rarely suspected at initial presentation. It is usually seen in association with complex malformations of the genitourinary tract.

**Case:**

A bifid insertion of the solitary ureter causing an uretero–VVF was associated with an obstructing transverse vaginal septum manifesting as menouria. Also seen were solitary crossed renal ectopia, bicornuate uterus and skeletal anomalies.

**Conclusion:**

In women with menouria without vaginal menstruation, pre-operative evaluation to detect an obstructive vaginal anomaly and unusual uretero–vesicovaginal fistulous communications is necessary before surgical intervention.

## CASE REPORT

A 33-year-old sexually inactive woman was referred for continuous dribbling of urine of 6 years duration.

From her 14^th^ year, she reported cyclical hematuria with severe suprapubic pain. She required no sanitary protection. However, she assumed this to be normal menstruation. She sought medical advice in her 16^th^ year and was advised no active intervention. She had recurrent episodes of urinary tract infection requiring antibiotic therapy since her 8^th^ year but did not experience urinary incontinence.

She developed acute urinary retention at 27 years. Ultrasound imaging showed hematocolpos of 15 × 9.7 cm, with a crossed right renal ectopia. As per records, her vagina was occluded by a thick septum (2.5 cm), which was excised, and approximately 1000 ml of pus was drained. She developed continuous urinary leakage along with normal voiding in the immediate post-operative period. Subsequently, her dysmenorrhea was relieved and menstruation occurred through the vagina.

She was referred to our center 6 years after the onset of continuous urinary leakage and was evaluated further. Physical examination revealed scoliosis. No other anomalies (systemic or somatic) were detected.

Intravenous urogram revealed a solitary crossed renal ectopia with mild hydroureteronephrosis. At examination under anesthesia (EUA), the lower vagina was stenosed. Cystoscopy and methylene blue (MB) test revealed a vesicovaginal fistula (VVF) proximal to the bladder neck on the left. The ureteric orifice was not visualized separately. The initial impression was that of a recurrent stenosis of the vagina with VVF, probably congenital, which explained the cyclical hematuria when there was an obstructing transverse vaginal septum.

Releasing incisions were made in the stenotic lower third of the vagina, which exposed the normal upper vagina and cervix. Vaginal repair of the fistula with Martius flap was carried out in order to improve the local vascularity in view of the vaginal stenosis and scarring that could have possibly compromised local vascularity

**Figure 1:**
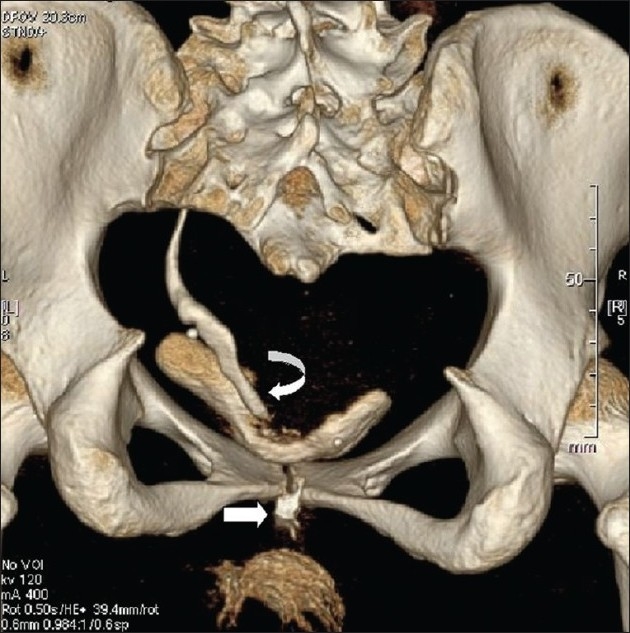
Three-dimensional reconstructed computed tomography image (posterior veiw); curved arrow showing insertion of the ureter and block arrow showing vaginal leak of contrast

Urinary leakage recurred on the 3^rd^ post-operative day onwards. She returned for further evaluation after a year. A computed tomography (CT) urogram and magnetic resonance urogram performed on the same day showed a right solitary kidney, the ureter crossed to the left (retrocavally at L 3 vertebra) and opening into the bladder just above the fornix with contrast (non-ionic iodinated used for CT) leak into the vagina and contrast opacification of the bladder [Figure 1]. Associated L 5 hemivertebra with scoliosis was seen.

**Figure 2:**
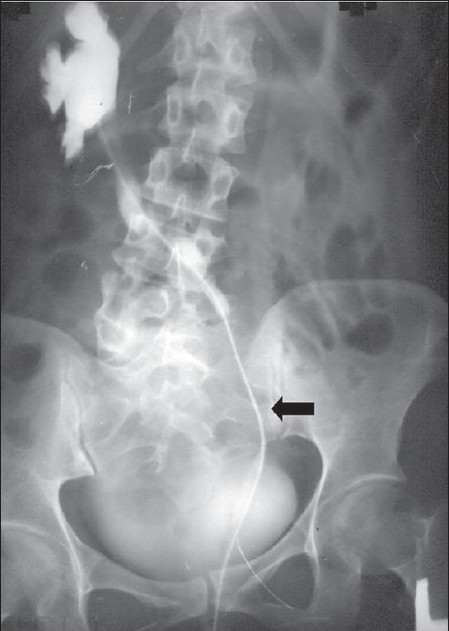
Arrow showing catheter in the ureter

Repeat EUA showed the fistula in the anterior vaginal wall toward the left. Retrograde cannulation and injection of MB showed efflux through the defect in the bladder base proximal to the bladder neck toward the left, as seen through a cystoscope. Further, the catheter went across this area without resistance and contrast study confirmed it to be retrograde pyelogram revealing the kidney on the right side [Figure 2].

Subsequently, she underwent ureteric reimplantation and transvesical closure of the VVF tract. Bicornuate uterus with a rudimentary right horn and pelvic endometriosis was also seen during surgery. Urinary incontinence recurred from the 20^th^ day following surgery.

A residual VVF was confirmed and repaired vaginally 4 months later, which relieved her of urinary leakage and she remained continent.

## DISCUSSION

In retrospect, our patient was diagnosed to have a congenital VVF with a variant of mesonephric duct-induced müllerian deformity type II anomaly.[1] She had a solitary crossed renal ectopia, bicornuate uterus with a rudimentary right horn and associated distal vaginal transverse septum.

Congenital VVF is a rare condition with less than 20 cases reported to date.

This patient presented to us with continuous urinary leakage with a probable diagnosis of VVF. Considering the history of cyclical hematuria before excision of the obstructing transverse vaginal septum, the VVF should be considered to be a congenital VVF.

We hypothesize that due to the obstructing low transverse septum, the vaginal collection of the menstrual blood found its way through the congenital VVF (path of least resistance) resulting in cyclical hematuria without vaginal menstruation.

Because of the obstructing vaginal septum, before the age of menarche there was no manifestation of urinary leak even though there was a congenital VVF. Urinary leak manifested only after the obstructing septum was excised. If there was no obstructive septum, this patient would have presented with urinary dribbling since childhood.

Persistent urinary leak following the first VVF repair, however, led us to investigate this patient further. Retrograde catheterization detected a previously unsuspected bifid communication between the bladder, vagina and the solitary ureter.

**Figure 3:**
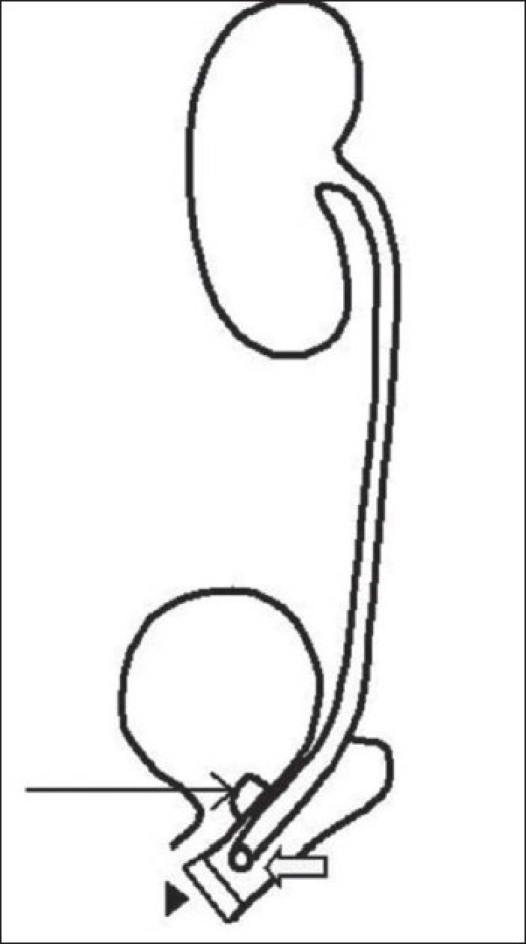
Line diagram depicting the bifid communication of the fistula. Line arrow depicting the fistulous opening above the bladder neck, block arrow depicting the vaginal communication of the solitary ureter and arrowhead depicting the obstructing vaginal septum

The uretero–vesicovaginal communication can be explained in two ways. The communication could have been a congenital bifid communication [Figure 3] or an iatrogenic communication. An iatrogenic uretero–vesicovaginal communication could have been caused due to close proximity of the ureteric orifice and the congenital VVF, the first attempted VVF repair converting this into a single common channel.

Failure to detect the entity of uretero–vesicovaginal communication and its intricacy during the initial evaluation probably led to the recurrence of the fistula following the first VVF repair.

An obstructive vaginal septum will conceal a congenital VVF and manifest as cyclical hematuria at menarche. Excision of the septum in this case unmasked the VVF and manifested as urinary leakage.

Similarly, urinary incontinence occurred only after the obstructing vaginal septum was excised in a patient who had a bizarre communication between the ectopic ureter and the right uterine cervix of an uterine didelphys. The ectopic ureter opened into the bladder neck and its proximal end communicated through a duct-like structure with the right uterine cervix.[2]

Congenital VVF has been commonly detected when menouria is associated with an obstructing vaginal septum.[2–5] Various types of unusual congenital VVF have been described in the reports.[2,4,6]

Obstructive genital lesions, although innocuous by themselves, have sexual, reproductive and menstrual sequelae as seen in our patient who developed endometriosis possibly due to retrograde menstruation. A transverse vaginal septum (American fertility society class IIA anomaly) is more common in the upper 1/3^rd^ of the vagina, but may occur at any level in the vagina, and 14% of the septae are seen in the lower vagina.[7]

Among women with unilateral renal agenesis, mullerian anomalies are seen in 25–50% and musculoskeletal defects in 14%.[1] These associations were also seen in this patient.

A history of cyclical hematuria (menouria) without vaginal menstruation should alert the clinician to the possibility of obstructive vaginal septum with congenital VVF. Considering this rare entity, imaging and contrast studies are warranted to delineate the unique communications that are possible between the genital and the urinary tracts. Although anatomic delineation of the fistula was possible with magnetic resonance imaging, the unique defect in our case was detected only on retrograde cannulation (catheterization). The vaginal 3-swab test of Moir is typically used to differentiate a VVF from a uretero vaginal fistula and was not performed in this case as the patient had a stenotic vaginal introitus at her first visit to our institution and also as she was unwilling for vaginal manipulation because she was sexually inactive. This test would have helped to diagnose the vesicovaginal component of the uretero–vesicovaginal communication. However, the unusual uretero–vesical component would have remained undiagnosed.

This report highlights the diagnostic and therapeutic challenge that a congenital VVF with obstructing transverse vaginal septum and subsequent recognition uretero–vesicovaginal communication can pose to a clinician. This case highlights the intricacies with reference to diagnosis and management of such cases.

Increasing awareness of this rare entity will have a positive impact in future recognition and a more meticulous evaluation before any surgical intervention in patients with complex genitourinary anomalies will decrease the morbidity and improve the quality of life of the patient.

It is therefore essential to review the lessons learnt from this case. An awareness of the rare possibility of a congenital VVF if there is a history of cyclical menouria is necessary. The presence of a VVF does not rule out other unusual uretero–vesicovaginal communications. In the absence of vaginal menstruation, an exploration for an obstructive vaginal anomaly is essential and it is imperative to identify the ureteric orifice before undertaking any surgical intervention.
